# Chloroplast Degradation: Multiple Routes Into the Vacuole

**DOI:** 10.3389/fpls.2019.00359

**Published:** 2019-03-26

**Authors:** Xiaohong Zhuang, Liwen Jiang

**Affiliations:** ^1^School of Life Sciences, Centre for Cell & Developmental Biology and State Key Laboratory of Agrobiotechnology, The Chinese University of Hong Kong, Hong Kong, China; ^2^The Chinese University of Hong Kong Shenzhen Research Institute, Shenzhen, China

**Keywords:** chlorophagy, autophagy, vacuole, chloroplast, chloroplast receptor

## Abstract

Chloroplasts provide energy for all plants by producing sugar during photosynthesis. To adapt to various environmental and developmental cues, plants have developed specific strategies to control chloroplast homeostasis in plant cells, including chloroplast degradation during leaf senescence and the transition of chloroplasts into other types of plastids during the day-night cycle. In recent years, autophagy has emerged as an essential mechanism for selective degradation of chloroplast materials (also known as chlorophagy) in the vacuole. Different types of membrane structures have been implicated to involve in the delivery of distinct chloroplast contents. Here we provide a current overview on chlorophagy and discuss the possible chloroplast receptors and upstream signals in this process.

## Introduction

The chloroplast, a well-known plastid found in all photosynthetic plant cells, is the central organelle providing plants with foods and energy in the form of sugar or starch by photosynthesis (Jarvis and Lopez-Juez, 2014). Chloroplast turnover plays a critical role in plastid transition (e.g., proplastid to chloroplast) and nutrient mobilization (e.g., carbon and nitrogen) (Siqueira et al., 2018). Upon different stress conditions, chloroplasts may be damaged and produce toxic ROS or stress signals which are detrimental to the plant growth. To cope with a variety of internal or external stresses, plants carry out leaf senescence via selective degradation of chloroplasts to avoid the accumulation of toxic ROS, thus placing a significance of efficient chloroplast turnover under stress conditions (Xie et al., 2015; Izumi and Nakamura, 2018; Nakamura and Izumi, 2018; Otegui, 2018; Soto-Burgos et al., 2018). Recent evidence suggests that chloroplast materials are sequestered into multiple types of subcellular structures for their delivery into the lytic vacuole. Novel insights into our understanding of chloroplast turnover have been obtained by recent studies on the relationship between chloroplast degradation and autophagy, a self-eating process conserved in all eukaryotic cells (Liu and Bassham, 2012). The accelerated leaf senescence observed in most autophagy-related (ATG) mutants suggests that autophagy might function as a strategy for carbon and nitrogen remobilization to the sink tissues by facilitating chloroplast degradation in the source tissues.

Three types of autophagy have been defined so far, including chaperone-mediated autophagy, macroautophagy and microautophagy (Mizushima and Komatsu, 2011). Chaperone-mediated autophagy, which depends on chaperone HSC70 and co-chaperones, has been reported in mamalian cells but not in yeast and plants (Mizushima and Komatsu, 2011). Macroautophagy occurs with the formation of a unique double membrane structure termed an autophagosome for the delivery of the cargos into the lysosomes/vacuole, and utilizes molecular machinery termed as ATG genes to generate the autophagosome. During autophagosome formation, an isolation membrane, named phagophore, engulfs and encloses the cargos to become a double membrane structure (Liu and Bassham, 2012). By contrast, during microautophagy, cargos are directly evaginated into the vacuole lumen by the vacuole membrane. Of note, microautophagy can be either ATG-dependent or ATG-independent (Mijaljica et al., 2011; Oku et al., 2017). These different types of autophagy have been implicated in cargo selectivity to facilitate the bulk or specific degradation of the target cargos under different conditions. In plant cells, excellent reviews have implicated that both macroautophagy and microautophagy pathways contribute to chloroplast degradation, and exhibit cargo specificity under different types of conditions (e.g., leaf senescence, carbon starvation or high light stress) by forming various types of structures (Xie et al., 2015; Izumi and Nakamura, 2018; Nakamura and Izumi, 2018; Otegui, 2018; Soto-Burgos et al., 2018). Here, we aim to compare these different pathways for the selective degradation of chloroplasts (here termed as chlorophagy), with an emphasis on the possible chloroplast receptors and related signals in this process.

## Macroautophagy-Like Degradation of Chloroplasts

**FIGURE 1:**
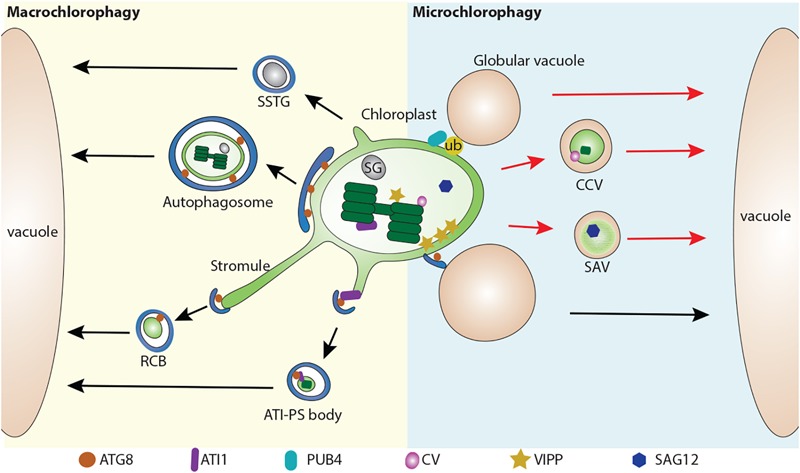
Multiple pathways for chloroplast degradation. Partial or whole chloroplast contents are sequestered into various types of compartments for degradation. In a macroautophagy-like (Macro chlorophagy, left), Rubisco-containing body (RCB) (Ishida et al., 2008), ATI1-GFP Labels Plastid-Associated Body (ATI-PS body) (Michaeli et al., 2014), and small starch granule-like structure (SSTG) (Wang Y. et al., 2013), as well as entire chloroplast (Izumi et al., 2017) are sequestered by a autophagosome. In a microautophagy-like (Micro chlorophagy, right) pathway, CV-containing vesicle (CCV) (Wang and Blumwald, 2014), senescence-associated vacuole (SAV) (Otegui et al., 2005), globular vacuole (Woodson et al., 2015) as well as direct vacuolar invagination of entire chloroplast (Nakamura et al., 2018) have been reported for the degradation of chloroplast contents. These pathways can be either ATG-dependent (arrows in black color) or ATG-independent (arrows in red color). ATG8, Autophagy-related protein 8; ATI1, ATG8 interacting protein 1; ATI-PS body, ATI1-GFP Labels Plastid-Associated Body; CV, CHLOROPLAST VESICULATION; CCV, CV-containing vesicles; RCB, Rubisco-containing body; PUB4, PLANT U-BOX 4; SAG12, Senescence-associated gene 12; SSTG, small starch granule-like structure; SG, starch granule; SAV, senescence-associated vacuole; VIPP, VESICLE INDUCING PROTEIN IN PLASTID1.

A macroautophagy-like process for either partial or whole chloroplast degradation utilizes the formation of autophagosomal structures and requires ATG proteins (Wada et al., 2009). Among these ATG proteins, ATG8 is widely used as an autophagosomal marker to label the autophagosomal structures (Yoshimoto et al., 2004; Contento et al., 2005; Zhuang et al., 2013, 2017, 2018; Spitzer et al., 2015). So far, several types of macroautophagy-related structures have been reported, including the Rubisco-containing body (RCB) (Ishida et al., 2008), the ATI1-GFP Labels Plastid-Associated Body (ATI-PS body) (Michaeli et al., 2014), and small starch granule-like structures (SSTG) (Wang Y. et al., 2013; Figure 1).

### RCB Pathway

The first observation of a macroautophagy-like process for chloroplast degradation came from the detection of Rubisco in small spherical bodies (around 1 μm) in both the cytoplasm and vacuole in wheat leaves, subsequently referred as RCBs (Chiba et al., 2003). Autophagosome-like double membranes are detected around these bodies (Figure 1). Another study using live-cell imaging to track the fate of RCBs (indicated by GFP-labeled Rubisco) in Arabidopsis and rice leaves demonstrated that the production of RCBs requires ATG genes including ATG5 or ATG7 (Ishida et al., 2008). Moreover, RCB fluorescent signals co-localize with the autophagosomal marker GFP-ATG8. This provides direct evidence that chloroplast proteins are degraded via autophagy by forming the RCBs and subsequent sequestration into autophagosomal structures. In addition, RCBs are highly induced by carbon deprivation and darkness, suggesting a role for autophagy in leaf carbon homeostasis by the degradation of chloroplast proteins via RCBs. In support of this, it has been observed that RCB numbers are increased in starchless mutants (Izumi et al., 2010, 2013). Therefore, RCBs represent a typical type of autophagic structure for chloroplast turnover.

It appears that the formation of RCBs is closely related to the extending stromule (Figure 1). Intriguingly, in the autophagy defective mutant *atg5*, many stromules are labeled by stroma-targeted GFP, suggesting that RCBs are released from the extending stromules (Ishida et al., 2008). In addition, stromules are highly induced by different abiotic and biotic signals, such as exposure to ROS or high sucrose/glucose conditions (Brunkard et al., 2015; Hanson and Hines, 2018). Such plasticity in chloroplasts by forming extending stromules in response to diverse stimuli might provide an efficient way for the removal of chloroplast materials. By GFP labeling in the confocal microscope and immunolocalization in transmission electron microscope (TEM), it was found that only Rubisco, but not chlorophyll, is detected in the stromules (Kwok and Hanson, 2004; Holzinger et al., 2008). This explains why thylakoid membranes are not detected in the RCBs. Intriguingly, extended stromules were also observed in a mutant defective in the Endosomal sorting complex required for transport (ESCRT) protein, CHMP1 (Spitzer et al., 2015). A primary role for the ESCRT complex in multivesicular body (MVB) biogenesis and endosomal sorting has been well characterized in plant cells (Gao et al., 2017). It is therefore proposed that CHMP1 might play an additional role in autophagosome maturation, thus a malfunction of CHMP1 might lead to an incomplete autophagosome, and the delay of RCB sequestration, resulting in the accumulation of phagophore-like structures and cytoplasmic RCBs (Spitzer et al., 2015).

### ATI-PS Body Pathway

Another type of autophagy-related degradation structure with a size around 1 μm, is termed the ATI-PS body, which is mediated by the ATI proteins (Michaeli et al., 2014). ATI proteins are plant-specific and no counterparts have been identified in non-plant species (Honig et al., 2012). They directly interact with ATG8 via the ATG8-interacting motif (AIM) and were previously identified as being distributed on the endoplasmic reticulum (ER). However, upon sugar starvation, ATI is detected on the outer membrane of chloroplast but not on stromules, precluding a similar origin to RCBs (Michaeli et al., 2014). Another major difference of ATI-PS bodies is that they contain different chloroplast cargos, including stromal, thylakoid and envelope proteins. Although the ATI-PS bodies are suggested to be derived from the chloroplast thylakoid, it raises a question as to how ATI, which is predicted to be a single transmembrane protein on the ER, is relocalized into the chloroplast lumen from the chloroplast outer membrane. Of note, the release of ATI-PS body from plastid is ATG-independent, but its delivery into the vacuole requires the ATG machinery (Michaeli et al., 2014).

### SSTG Pathway

Starch granules, which are wildly deposited in the chloroplast, serve as an essential carbon reservoir by converting starch into sugar (Malinova et al., 2018). A previous study revealed that starch contents are greatly decreased (about 70%) in *atg4a4b-1* upon exposure to the darkness (Izumi et al., 2010). In another study, electron microscopy has shown that a small starch granule-like structure (usually with a diameter of < 0.5 μm) was captured in the cytosol and sequestered into the autophagic bodies (Wang Y. et al., 2013). These SSGLs were clearly labeled by YFP-tagged granule-bound starch synthase I (GBSSI-YFP), a starch granule marker, and also colocalized with the autophagosome marker CFP-ATG8f. It also appears that the occurrence of stromules may contribute to the release of SSGL, which is supported by the detection of SSGL in the stromules by both confocal and electron microscopy (Wang Y. et al., 2013). Similar to RCBs and ATI-PS bodies, the number of vacuole-localized SSGLs was greatly reduced after blocking autophagic activity via ATG6 silencing, suggesting that the degradation of SSGLs are also ATG-dependent (Wang Y. et al., 2013).

### Whole Chloroplast Pathway

Previous study has shown that whole chloroplasts are delivered to vacuoles in individually darkened leaves which display accelerated senescence due to sugar starvation (Wada et al., 2009). A recent study has also showed that whole chloroplasts can be targeted for degradation by autophagy upon exposure to UV light (Izumi et al., 2017). After UV light exposure, the autophagosomal membrane, as labeled by GFP-ATG8a, captures the whole chloroplast and encloses it into a completed autophagosome. These autophagosomal structures are much larger than the previously described chlorophagy-related structures, and can be readily detected in the vacuole as well. Moreover, plants lacking autophagic activity have less vacuolar delivery of these UV light-triggered structures into the vacuole and display a higher sensitivity to UV-B exposure. It is suggested that the invagination of the entire chloroplast is different from the RCB pathway, as it occurs independent of the activation of the RCB pathway. However, in both *atg5* and *atg7* mutants, damaged chloroplasts with extended stromules also accumulated upon UV-B exposure (Izumi et al., 2017), raising the possibility that stromule formation might contribute to whole chloroplast degradation as well.

## Microautophagy-Like Degradation of Chloroplasts

In comparison to macroautophagy, microautophagy mediates the degradation of chloroplast by direct invagination of the chloroplast contents via the vacuole membrane (Figure 1). A recent study showed that high-intensity light (HL) will trigger chloroplast envelope damage and lead to chloroplast swelling (Nakamura et al., 2018). In addition, overexpression of VESICLE INDUCING PROTEIN IN PLASTID1 (VIPP1), a protein that regulates chloroplast envelope integrity, causes the formation of abnormal swollen chloroplasts (Nakamura et al., 2018). These swollen chloroplasts are detected in the vacuole under HL or VIPP1 overexpression conditions. Interestingly, it appears that the swollen chloroplasts are initially recognized by the ATG8-containing structures prior to vacuole invagination. Furthermore, confocal imaging analysis showed that the entire swollen chloroplast is directly engulfed by the GFP-δTIP labeled tonoplast into the vacuole, while this process is absent in the *atg5* mutant, supporting the involvement of an ATG-dependent microautophagy-like process. The role of the ATG8-labeled structure is suggested to serve as a selective platform for chloroplast recognition via an interaction between the chlorophagy receptor(s) and ATG8, in a manner similar to that for the ATI-PS body. Alternatively, the formation of ATG8-sac structures may facilitate the deposition of the cap-like structure at the chloroplast to control the docking and fusion between the chloroplast membrane and the tonoplast, followed by the release of the chloroplast contents into the vacuole lumen.

Other studies have also reported other types of structures for the execution of chloroplast degradation in a microautophagy-like manner, although they were not initially defined as a microautophagy-like process. For example, senescence-associated vacuoles (SAVs), which are characterized by a senescence-induced cysteine protease Senescence-associated gene 12 (SAG12), were identified as a distinct type of lytic compartment during leaf senescence (Otegui et al., 2005). SAVs display similar characteristics to the lytic vacuole as they are stained by LysoTracker red or neutral red, although they lack the tonoplast marker γ-TIP (Otegui et al., 2005). Another study showed that isolated SAVs contain stromal proteins including Rubisco and glutamine synthetase, but lack thylakoid proteins (Martinez et al., 2008). It was claimed that SAVs are still formed in the *atg7* mutant, thus representing a separate pathway for chloroplast turnover (Otegui et al., 2005). However, in another study, by targeting chloroplasts with CHLOROPLAST VESICULATION (CV), it is shown that CV-containing vesicles (CCVs) are accumulated under abiotic stress and downregulated by cytokinin (Wang and Blumwald, 2014). Chloroplast materials including stroma, envelope, and thylakoid proteins are identified in these CCVs. The authors also showed that the formation of CCVs is autophagy-independent and is separate from the autophagosome marker GFP-ATG8a. Instead, bimolecular fluorescence complementation (BiFC) assays showed that CV interact with PsbO protein, a component of photosystem II complex on the thylakoid membrane, suggesting that CCVs may arise from within the chloroplast. Recently, it has also been shown that CV is regulated by a NAC transcription factor RD26 (Kamranfar et al., 2018), which also regulates ABA-related genes (Zhang and Gan, 2012), implying a feedback regulation between ABA signaling and chloroplast degradation via the CCVs. However, lacking an evident observation of the intermediate structures for the formation of SAVs and the CCVs renders their origins obscure.

## Chlorophagy-Related Membrane Receptors in Plants

A critical question in regarding to chlorophagy is how selectivity for chloroplast contents is executed during this process. Specific autophagic receptors are known to function in different types of autophagy, such as mitophagy, pexophagy and ER-phagy (Zaffagnini and Martens, 2016). The organelle components are recognized by autophagic receptors for docking to the autophagosomal structure or the vacuole membrane. So far, the autophagic receptors identified usually contain a canonical AIM, and a specific cargo interacting domain, and are either ubiquitin-dependent or ubiquitin-independent (Zaffagnini and Martens, 2016).

However, with respect to the chloroplast being specific for plant cells, it is not surprising that plant-unique receptors might be involved in chlorophagy. ATI1/2 represents a type of plant-specific receptor for chlorophagy which interacts with ATG8 upon sugar starvation (Michaeli et al., 2014). Several chloroplast-localized proteins have also been found to interact with ATI by the split ubiquitin yeast two-hybrid assay, and it was suggested that these proteins are sequestered into the ATI-PS body via ATI interaction (Michaeli et al., 2014). However, ATI is localized on the ER under normal conditions and how the sugar signal activates the recruitment of ATI onto the outer membrane of the chloroplast remains unknown (Honig et al., 2012). An extensive supply of lipid precursors from the endoplasmic reticulum (ER) to the chloroplast have been implicated to be mediated by a group of TRIGALACTOSYLDIACYLGLYCEROL (TGD) proteins, and only TGD4 has been shown to be distributed on the outer membrane of the chloroplast (Fan et al., 2015). One possibility would be that ATI is translocated onto the chloroplast via ER-chloroplast contact sites, so that it can be mobilized via lipid exchange to aid the initiation of the ATI-PS body (Zhuang et al., 2016).

**FIGURE 2:**
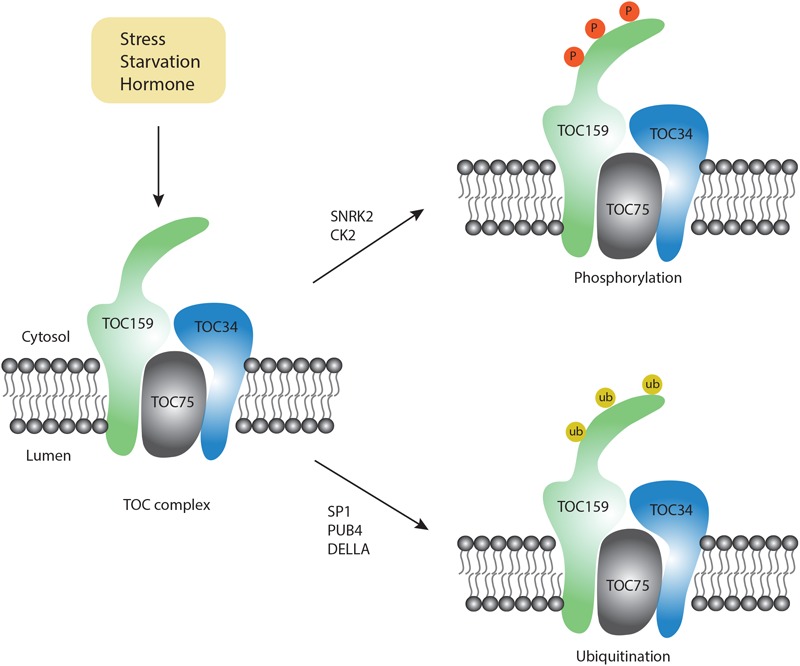
Post-modification on the TOC complex. Upon exposure to different conditions, two types of modifications occur on the TOC complex, including phosphorylation (orange color) and ubiquitination (yellow color).

In addition to ATI1/2, there are no proteins on the plant chloroplast surface that have been experimentally tested to have a similar function during chlorophagy. Using a Bioinformatic tool for prediction of proteins that may interact with ATG8 (Kalvari et al., 2014), and based on the available experimental information of the chloroplast outer membrane proteins in Arabidopsis (Lee et al., 2014), a set of predicted AIM-containing proteins in Arabidopsis is listed in Table 1, including TOC159, TOC75 and TOC33. TOC159, TOC75, and TOC33 are inserted into the outer membrane of chloroplast, forming the chloroplast protein TOC import complex for the import of chloroplast proteins (Lee et al., 2014). Particularly, post-modifications of the TOC complex under various induction conditions have been reported (Agne et al., 2010; Ling et al., 2012; Wang P. et al., 2013; Woodson et al., 2015; Shanmugabalaji et al., 2018; Figure 2). In addition to the TOC complex, potential AIMs were also identified in the outer envelope protein (OEP) complex, which serves as an alternative pathway for chloroplast protein import (Lee et al., 2014). OEP7 and OEP9 have been shown to function together with heat shock protein Hsp17.8 and AKR2A cofactors in targeting membrane proteins to plastid outer membranes under normal physiological conditions (Niehaus et al., 2014; Kim et al., 2015). To enable the efficient recognition by the autophagosomal or vacuolar membrane, it is possible that these chloroplast outer membrane proteins might serve as receptors by binding to the ATG or non-ATG chlorophagy regulators. However, since these plant-specific chloroplast proteins lack counterparts in either yeast or mammalian cells, more effort will be required in the future to validate the predicted AIMs and their possible roles in different types of chloroplast degradation pathways.

**Table 1:** Predicted chloroplast outer membrane proteins containing the ATG8-interacting motif in Arabidopsis.

Name	Gene	ATG8-interacting motif	Position from (aa)	Position to (aa)
Toc33	At1g02280	EFFGKL	24	29
Toc34	At5g05000	REWIGI	8	13
		NLFNKI	237	242
Toc64/OEP64	At3g17970	NLWVLL	7	12
Toc75I	At1g35860	YSFANV	55	60
Toc75III	At3g4674	GMFEKV	223	228
Toc75-IV	At4g09080	/	/	/
Toc75-V/OEP80	At5g19620	/	/	/
Toc159	At4g02510	GEFEPV	286	291
		KTYASV	23	28
		YRYRYL	1265	1270
		SIYKSI	1510	1515
OEP7	At3g52420	LGWLAI	19	24
OEP9	At1g16000	/	/	/
OEP61	At5g21990	ADFARI	24	29
OEP21A	At1g20816	EMFEKV	138	143
OEP21B	At1g76405	EMFDKV	138	143
OEP24A	At1g45170	PSFNGL	43	48
		PGFFII	55	60
		LKYTYV	126	131
OEP24B	At5g42960	GSFI V	57	62
OEP37	At2g43950	LGWASL	298	303
PDV1	At5g53280	PGYVFI	62	67
PDV2	At2g16070	KDFEVL	130	135
Cytochromeb5	At1g26340	DCWVVI	21	26
		KQYWVV	112	117
/	At4g16070	DSWTGI	412	417
/	At4g27610	PNWILI	22	27
/	At5g11250	FSYDAL	481	486
		IGFFTL	14	19
		RDFDGL	234	239
		IIYSGL	1162	1167
TGD4	AT3g06960	PSFSPI	64	69
		AVWPGL	193	198

*^∗^The order of the predicted motifs are based on the score obtained on the iLIR web tool (http://repeat.biol.ucy.ac.cy/iLIR/). aa, amino acid.*

## Possible Signals and Post-Translational Modification for the Chloroplast Proteins During Chlorophagy

Although chlorophagy is highly induced by various stress conditions, the molecular signals for chlorophagy activation remain unknown. Notably, RCB formation requires stromule formation, which is highly inducible under starvation and stress conditions (Ishida et al., 2008). For example, it has been reported that stromule number is increased upon ABA treatment (Gray et al., 2012). On the other hand, ABA modulates the activity of downstream factors like ABI and P2CC, which further activate the SnRK2 kinase complex (Soon et al., 2012). Particularly, the SnRK complex also serves as a critical upstream regulator for autophagic activation by phosphorylation of a set of ATG proteins, e.g., ATG1 (Soto-Burgos et al., 2018). Although there is no direct evidence to link ABA signaling with autophagy, a recent study using quantitative phosphoproteomics showed that phosphorylation of TOC159 homologs TOC132 and TOC120 occurs in an ABA-dependent manner only in the SnRK2-deficient mutant background (Wang P. et al., 2013). Furthermore, *in vitro* phosphorylation assays have demonstrated that TOC159 might be directly phosphorylated by recombinant SnRK2 proteins (Wang P. et al., 2013). In another study, phosphorylation of recombinant A-domain of TOC159 was also observed when incubated with casein kinase 2 (CK2)-like proteins (Agne et al., 2010), an evolutionarily conserved serine/threonine protein kinase to function in circadian clock regulation. Recently, it was shown that CK2 also participates in the ABA-activated SnRK2 signaling pathway (Vilela et al., 2015). Therefore, it is possible that SnRK2- or CK2- dependent phosphorylation of chloroplast membrane proteins or the ATG proteins activates chlorophagy in coordination with the ABA signaling pathway. It will be interesting to test whether the phosphorylation of these chloroplast membrane-associated proteins would serve as a signal to recruit the ATG machinery onto the chloroplast membrane.

In addition to phosphorylation, another type of essential post-translational modification, ubiquitination, might provide the specificity for the selection of chloroplast cargos. Emerging evidence from both yeast and mammalian cells supports a close interplay between ubiquitination and autophagy (Shaid et al., 2013). In plants, it is well known that ubiquitination plays a critical role in chloroplast biogenesis during plastid developmental transitions (e.g., proplastid to chloroplast in seed germination). In a recent study, it was reported that during germination, the DELLA proteins promote TOC159 degradation via ubiquitination to modulate proplastid to chloroplast transition during early plant development (Shanmugabalaji et al., 2018). Although it is claimed that ubiquitinated TOC159 is degraded via the proteasome pathway, whether ubiquitinated TOC159 may be targeted by chlorophagy awaits to be determined. Of note, TOC159 has previously been identified as a substrate of another E3 ligase SP1, which is distributed on the outer membrane of the chloroplast to regulate chloroplast biogenesis (Ling et al., 2012). Interestingly, MUL1, the closest homolog of SP1 in humans, has been shown to regulate the ULK1 (ATG1 homolog in human) activity and mitophagy negatively (Li et al., 2015; Rojansky et al., 2016). Upon selenite treatment, it was observed that ULK1 translocates to mitochondria to interact with MUL1. In plants, SP1 has been detected on peroxisomes, mitochondria as well as chloroplasts (Pan and Hu, 2018). Additionally, it was reported that Parkin, a E3 ubiquitin ligase in mammalian cells, interacts with the mitochondrial outer membrane protein VDAC to regulate its ubiquitination for the targeting of mitochondria during mitophagy (Geisler et al., 2010). Hence, it is possible that SP1 might play an additional role during chlorophagy and future work is needed to identify potential SP1 substrates during chlorophagy.

Recently, in a screening for a suppressor of *fc2*-triggered chloroplast degradation, a E3 ligase PLANT U-BOX 4 (PUB4) was identified (Woodson et al., 2015). It was implied that PUB4 functions in chloroplast turnover via ubiquitination of the chloroplast proteins during dark-to-light transitions against ROS. Of note, loss-of-function of the chloroplast protein import machinery (TOC33 and TOC159), also suppresses *fc2-*triggered chloroplast degradation, implying a coordination between the chloroplast import machinery and degradation. It appears that a *pub4* mutant exhibits less sensitivity to carbon starvation than the *atg* mutant, thus the authors claimed that the PUB4-mediated chloroplast degradation is likely to be independent of autophagy. However, during the PUB4-dependent chloroplast degradation process, it was observed that the damaged chloroplast directly fuses with a globular vacuole, which is quite similar to the microautophagy pathway through direct vacuolar invagination. It should be pointed out that the ATG machinery is dispensable during microautophagy and diverse molecular machineries are identified in other species. For instance, the ESCRT machinery but not the ATG machinery has been demonstrated to participate in the incorporation of cytoplasmic proteins into the vacuole (Oku et al., 2017).

## Perspective

Chloroplast homeostasis is critical for efficient nutrient recycling and remobilization. A significant role for autophagy in senescent leaves is to avoid the accumulation of toxic products from the chloroplast by removing the damaged or excessive chloroplast contents. For instance, chloroplasts produce ROS and stress hormones (e.g., salicylic acid and ABA precursor), both of which can alter nuclear gene expression and accelerate leaf senescence (Yoshimoto et al., 2009; Schippers et al., 2015). Thus, degradation of chloroplasts by autophagy may promote cell survival, and contribute to the natural turnover of aging chloroplasts to overcome early leaf senescence and cell death. However, so far, little is known about how chlorophagy is regulated and how the selectivity of chloroplast materials is achieved. We anticipate that more efforts will be put forward in future toward the identification of novel chloroplast regulators to link with the ATG machinery. In particular, identification of the distinct chlorophagy receptors for different pathways as well as their interaction network should provide more insights into how these different pathways are coordinated for chloroplast turnover. In addition, although previous studies visualizing chlorophagy-related structures have mainly relied on 2D transmission electron microscopy images or confocal microscopy imaging, details on the intermediate structures are still missing. Outstanding questions are as follows: Why are there so many types of pathways/structures and are they related? What happens if a pathway/structure is inhibited? How are vesicles initiated within the double-membrane chloroplast (e.g., ATI-PS)? How does the outer membrane of chloroplast fuse with the vacuole? Given the complexity of chloroplast morphology, a combination of advanced techniques such as 3D electron microscopy and dynamic imaging should provide more insights into chlorophagy at the cellular level in the future.

## Author Contributions

XZ designed the concept and the organization of the manuscript. XZ and LJ wrote and edited the manuscript.

## Conflict of Interest Statement

The authors declare that the research was conducted in the absence of any commercial or financial relationships that could be construed as a potential conflict of interest.
